# Pulmonary Epithelial Integrity in Children: Relationship to Ambient Ozone Exposure and Swimming Pool Attendance

**DOI:** 10.1289/ehp.7027

**Published:** 2004-09-13

**Authors:** Birgitta Json Lagerkvist, Alfred Bernard, Anders Blomberg, Erik Bergstrom, Bertil Forsberg, Karin Holmstrom, Kjell Karp, Nils-Goran Lundstrom, Bo Segerstedt, Mona Svensson, Gunnar Nordberg

**Affiliations:** ^1^Environmental and Occupational Medicine, Department of Public Health and Clinical Medicine, Umea University, Umea, Sweden; ^2^Unit of Industrial Toxicology, Catholic University of Louvain, Brussels, Belgium; ^3^Respiratory Medicine and Allergy, Department of Public Health and Clinical Medicine; ^4^Paediatrics, Department of Clinical Sciences, and; ^5^Clinical Physiology, Department of Surgical and Peri-operative Sciences, Umea University, Umea, Sweden

**Keywords:** airway irritants, children, Clara cell protein (CC16), nitrogen trichloride, ozone, swimming pool

## Abstract

Airway irritants such as ozone are known to impair lung function and induce airway inflammation. Clara cell protein (CC16) is a small anti-inflammatory protein secreted by the nonciliated bronchiolar Clara cells. CC16 in serum has been proposed as a noninvasive and sensitive marker of lung epithelial injury. In this study, we used lung function and serum CC16 concentration to examine the pulmonary responses to ambient O_3_ exposure and swimming pool attendance. The measurements were made on 57 children 10–11 years of age before and after outdoor exercise for 2 hr. Individual O_3_ exposure was estimated as the total exposure dose between 0700 hr until the second blood sample was obtained (mean O_3_ concentration/m^3^ × hours). The maximal 1-hr value was 118 μg/m^3^ (59 ppb), and the individual exposure dose ranged between 352 and 914 μg/m^3^hr. These O_3_ levels did not cause any significant changes in mean serum CC16 concentrations before or after outdoor exercise, nor was any decrease in lung function detected. However, children who regularly visited chlorinated indoor swimming pools had significantly lower CC16 levels in serum than did nonswimming children both before and after exercise (respectively, 57 ± 2.4 and 53 ± 1.7 μg/L vs. 8.2 ± 2.8 and 8.0 ± 2.6 μg/L; *p* < 0.002). These results indicate that repeated exposure to chlorination by-products in the air of indoor swimming pools has adverse effects on the Clara cell function in children. A possible relation between such damage to Clara cells and pulmonary morbidity (e.g., asthma) should be further investigated.

Ozone is an important component of air pollution. Ground-level O_3_ in urban air is formed in a photochemical reaction between oxygen and nitrogen dioxide from fossil fuel emissions under the influence of sunlight and volatile hydrocarbons. Therefore, the O_3_ levels tend to be high in areas with high intensity of ultra-violet radiation and high emissions of NO_2_ from car traffic or industries using fossil fuels (American Thoracic Society 1996; de Marco et al. 2002).

Epidemiologic and controlled human studies as well as animal experiments on exposure to O_3_ have reported airway inflammation and/or a decrease in lung function at ambient concentrations [reviewed by Balmes (1993); Krishna et al. 1995]. Human experimental exposure to O_3_ has demonstrated a spectrum of acute airway responses. Among these are decrements in forced vital capacity (FVC) and forced expiratory volume in 1 sec (FEV_1_), increased airway resistance (Blomberg et al. 1999; Seal 1993), altered airway permeability, and antioxidant defenses (Blomberg et al. 1999; Mudway et al. 2001), as well as a neutrophilic airway inflammation (Schelegle et al. 1991; Stenfors et al. 2002). Most of these studies were short-term exposures on healthy adults. Acute lung function changes in children have been shown in field exposures. Associations between ambient O_3_ levels and reductions in FVC and FEV_1_ in children in summer camps and big cities have been shown in several studies (American Thoracic Society 1996; Kopp et al. 2000). Recently, short-term effects of O_3_ were observed in children as an increased frequency of emergency visits for asthma (Fauroux et al. 2000)

Repeated exposure to other environmental and occupational gases (e.g., sulfur dioxide and chlorine) also increase the risk of airway irritation and asthmalike symptoms (Olin et al. 2002). Several studies have shown that competitive swimmers have an increased prevalence of airway inflammation, bronchial hyperresponsiveness, and asthma (Helenius and Haahtela 2000; Potts 1996). This was attributed to inhalation of chlorine gas and its derivatives formed by chlorination of ammonia derived from organic matter in swimming pool water [e.g., nitrogen trichloride, trichloramine, or chlorine azide (NCl_3_)].

In recent years there has been a growing interest in noninvasive indicators as a means to detect early effects of air irritants (Bernard et al. 1992; Broeckaert et al. 1999). Several reports describe different lung-specific secretory proteins, which may be used to detect changes in the number of and/or integrity of epithelial secretory cells (Hermans and Bernard 1999). One of these, Clara cell protein (CC16), is a small, 16-kDa protein produced and secreted by the nonciliated bronchiolar Clara cells and detectable in serum. CC16 has antioxidant properties, and the levels in serum increase when lung epithelium permeability is adversely affected by air pollutants or other lung toxicants (Broeckaert et al. 1999; Hermans and Bernard 1996). On the other hand, reduced levels of CC16 in lung lavage fluid are described in several lung disorders (e.g., chronic bronchitis) and in smokers. This may be caused by a decrease in the production of CC16 depending on a decreased number of Clara cells (Hermans and Bernard 1999). In this study we validated CC16 and the lung surfactant proteins A, B, and D in blood as biomarkers of adverse pulmonary effects. The advantage of studying lung proteins in serum instead of in bronchoalveolar lavage, which has been commonly used to study inflammatory effects in the airways, is obvious. Blood samples are more easily obtained than is lung lavage. Besides, lung lavage is not a suitable method in studies on children.

The general aim of the present program, which was part of a European Union project (HELIOS), was to examine lung function and possible changes in the serum levels of CC16 in relation to ambient O_3_ exposure in Italy, France, Belgium, and Sweden (Bernard et al. 2003). The effects of exposure to other environmental factors (e.g., chlorine and its by-products) in swimming pools were also examined. The present study was conducted in Umea, a town in northern Sweden with low to moderate O_3_ levels, and was divided into four substudies. The first part was conducted on healthy adults in winter, when O_3_ levels were low, and the second part in the summer when O_3_ levels were known to be higher than in other seasons. Similar studies were then repeated in children. In the present report we describe the results from the summer study on children.

## Materials and Methods

The winter study on children, also the basis for the summer study, was conducted in November 2001; 139 children, 63 girls and 76 boys, from four primary schools were recruited. Children with a history of asthma or kidney disease were not included. The recruitment of school children was done according to the same protocol as in the study by Bernard et al. (2003) in Brussels. Lung function measurements were performed, and peripheral blood samples were obtained for analysis of CC16 in serum. In the present, summer, study, 57 of the children from the winter study were included, 56 Caucasian and one Chinese. The study was conducted in May 2002. The local ethics committee at Umea University approved the study protocol. A written informed consent was obtained from the parents.

The selection of the 57 children (33 boys and 24 girls) from the larger study in November 2001 was based on the results from the lung function tests and a questionnaire answered by the parents. Subjects who reported pollen allergy or childhood asthma and/or who had an FVC or FEV_1_ < 80% of the predicted value were not included, nor were children whose blood samples had difficulties or whose questionnaires were missing. The age (mean ± SD) of the participating children was 10.8 ± 0.4 years. Lung function testing and blood sampling (in November 2001) were repeated twice, before and after light exercise outdoors for 2 hr (range, 1.5–3.0 hr). The parents completed a questionnaire on, for example, current food intake, passive smoking, and airway illness since the larger, winter study. The participating children answered questions on outdoor activities and swimming pool attendance. Nearly 40% of the children were regular indoor pool visitors (i.e., they had visited an indoor pool for at least 1 hr/month during 6 months or longer). Sodium hypochlorite (1% chlorine) was used to disinfect the pool water. Based on swimming pool attendance according to the questionnaire, the children were divided into two subgroups, 34 non-pool visitors and 23 pool visitors.

We determined lung function parameters (e.g., FVC and FEV_1_) using a portable spirometer connected to a computerized data program (KoKo Spirometer and KoKo DigiDoser; Pulmonary Data Service Instrumentation, Inc., Louisville, KY, USA). The instruments were calibrated in the morning and after every 10th measurement. Changeable filter mouthpieces were purchased from Intramedic AB (Balsta, Sweden). One trained lung physiologist tested the lung function in all children. The tests were carried out in the standing position. The best reproducible flow/volume curves were used in the analysis. The computer program calculated the predicted normal values as a function of sex, age, height, and weight according to Polgar and Promadhat (1971).

Blood samples were obtained from the antecubital vein after local anesthesia with a cream or plaster (EMLA, AstraZeneca Ltd., Sodertalje, Sweden) immediately before (S1; four missing samples) and after (S2; three missing samples) the outdoor session. Two CC16 values were available for 20 pool visitors and 31 non-pool visitors. Blood (7.5 mL) was drawn in Sarstedt Monovette tubes for serum (Serum Z/9 mL, Sarstedt, Landskrona, Sweden). Each sample was allowed to clot for 1–2 hr at room temperature. After centrifugation at 3,000 rpm (within 2 hr after sampling), the serum was transferred to cryotubes and frozen at −80°C. These samples were then sent to the Industrial Toxicology Unit at the Catholic University of Louvain in Brussels for analysis. CC16 was determined by a latex immunoassay using rabbit anti-CC16 antibody (Dakopatts, Glostrup, Denmark) and also CC16 purified according to the standard in the laboratory (Bernard et al. 1992; Carbonelle et al. 2002). The assay has been validated by comparison with a monoclonal-antibody–based enzyme-linked immunosorbent assay (ELISA) (Hermans et al. 1998). All samples were run in duplicate at two different dilutions. The between- and within-run coefficients of variation ranged from 5 to 10%.

Outdoor O_3_ was monitored continuously at the university campus where the children spent time outdoors, using a Dasibi ultra-violet photometry ozone analyzer (model 1108; Dasibi Environmental Corporation, Glendale, CA, USA). O_3_ exposure was estimated as the total exposure of O_3_ between 0700 hr and the time the second blood sample was taken, between 1300 and 1600 hr (mean O_3_ concentration/m^3^ × number of hours). Because the children spent part of that time indoors (mean, 4 hr) and because it is known from other studies that indoor concentrations of O_3_ are lower than those outdoors (American Thoracic Society 1996), each individual’s exposure dose was estimated by assuming an exposure level of 50% of the mean outdoor O_3_ concentration during time spent indoors. This assessment was confirmed by measurement with passive diffusion samplers in the examination room. The filters were purchased from and analyzed at IVL Swedish Environmental Research Institute, Ltd. (Gothenburg, Sweden). The mean indoor O_3_ level during the study period was 40 μg/m^3^ (20 ppb).

The statistical program SPSS 11 (SPSS Inc., Chicago, IL, USA) was used for statistical analyses. Differences in FEV_1_ and CC16 before and after exercise and differences between groups were assessed with Student’s *t*-test, paired and unpaired. Pearson correlation tests were used for the analyses of correlations. A *p*-value < 0.05 was considered statistically significant.

## Results

The mean daytime outdoor O_3_ concentration in the days studied ranged from 77 to 116 μg/m^3^, and the maximal 1-hr value was 118 μg/m^3^. The estimated individual exposure dose varied from 352 to 914 μg/m^3^hr.

FEV_1_ was significantly higher after outdoor exercise than before in both children who had regularly attended chlorinated swimming pools and children not swimming (Table 1). These differences remained also if the percentages of the predicted FEV_1_ (FEV_1_% predicted) were compared. The mean measured FEV_1_ values varied between 91.2 and 93.0% of the predicted ones. There were no significant differences between pool visitors and non-pool visitors, when comparing FEV_1_% predicted either before (*p* = 0.43) or after exercise (*p* = 0.45, Student’s *t*-test), nor was there any significant difference in body mass index (BMI) between the two groups of children.

The mean ± SD serum concentrations of CC16 in non-pool visitors were 8.2 ± 2.8 μg/L before exercise and 8.0 ± 2.6 μg/L after exercise. The corresponding values in pool visitors were 5.7 ± 2.4 and 5.3 ± 1.7 μg/L (Table 2; range, 2.2–16.1 μg/L). The BMI was 18.5 ± 2.9 kg/m^2^. Only one pool visitor and three nonvisitors were exposed to passive smoke. There were no significant correlations between the serum CC16 levels and parental smoking or BMI. No significant differences were found between pre- and postexposure levels of serum CC16, nor did the time spent outdoors (mean, 6 hr) during the 2 days preceding the test day have any influence on the CC16 levels. However, the average CC16 levels in pool visitors both before (S1) and after (S2) exercise were lower than in non-pool visitors (*p* < 0.01) (Table 2). Twenty-two children regularly visited an indoor swimming pool for 1–35 hr/month (median, 4 hr/month). The children had been visiting indoor swimming pools regularly between 6 months to 10 years (median, 3 years). Only two children had been swimming since they were babies. No statistically significant relationship was found for attending a swimming pool during the last days before the test, probably because only seven children had attended indoor swimming pools the last 2 days before the test. In our study, we did not find any correlation between parental smoking and effects on the airways of the children or CC16 levels, possibly because only one pool visitor and three non-pool visitors were exposed to passive smoke.

**Figure 1:**
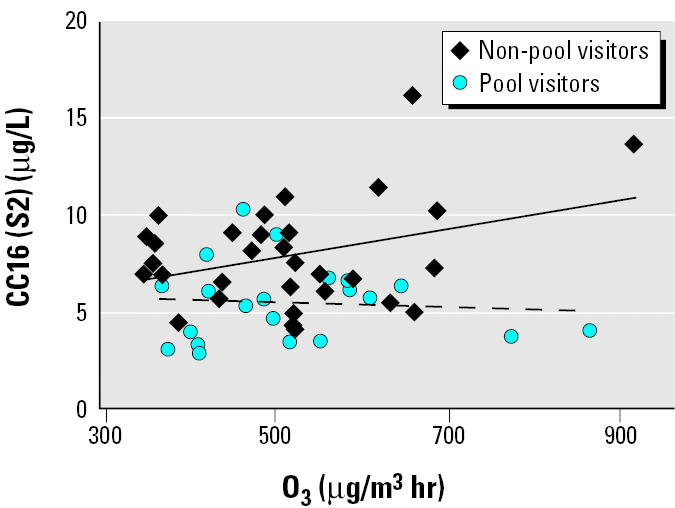
Correlation between the individual O_3_ exposure dose and serum CC16 concentration (μg/L) after 2 hr of outdoor exercise. The solid and dashed lines represent the correlation presented in Table 3: respectively, non-pool visitors and pool visitors.

The correlations between O_3_ exposure and CC16 levels before or after exercise outdoors were not statistically significant in the group as a whole. However, when CC16 after exercise (S2) was considered, there was a tendency toward a correlation in non-pool visitors after exercise (*p* < 0.06) (Table 3, Figure 1).

## Discussion

In this study, moderate O_3_ levels between 77 and 116 μg/m^3^ did not have any adverse effect on the lung function parameter FEV_1_ after 2 hr of outdoor exercise. In fact, the FEV_1_ was slightly increased at the second measurement. This could be an effect of better test performance after exercise than before. The ambient O_3_ levels in our study are also lower than those reported to affect lung function parameters at ambient O_3_ concentrations (Kinney et al. 1996; Nickmilder et al. 2003).

The serum CC16 levels found in this study did not correlate with BMI, a result that has been shown in other studies as well (Hermans et al. 1998). They were of the same magnitude as those recently reported in children of the same age in Belgium (Bernard et al. 2003; Carbonelle et al. 2002). In those studies, the serum levels of CC16 in children did not change significantly during swimming exercise. In the present study, we have compared the serum CC16 levels in pool visitors and in a control group not exposed to chlorination by-products and found significantly lower levels of serum CC16 in pool visitors suggesting adverse effects on Clara cells.

There were no significant differences between the levels of CC16 before and after outdoor exercise. Neither were there any statistically significant relationships between CC16 levels in serum and ambient O_3_ exposure, although a marginally significant tendency was found among nonswimmers (Figure 1). The lack of statistical significance may be due to the limited number of subjects and/or the O_3_ levels’ not being high enough to cause a response. In the present study, the O_3_ concentration was approximately one-fourth of the O_3_ concentration that recently was found to increase the serum CC16 levels in adult subjects (*n* = 22) exposed for 2 hr in an exposure chamber to 400 μg/m^3^ O_3_ (Blomberg et al. 2003). Another possible explanation for the lack of a clear relationship between serum CC16 and the O_3_ dose in the present study is that the time period between the measurements was not long enough to cause a measurable change in CC16 levels. There may also be an interference with diurnal variation not corrected for in the present study. Such a diurnal variation was indicated in a recent study on adults (*n* = 19) (Helleday et al. 2003). The reason why Bernard et al. (1997) did not find any significant variations in serum CC16 between 0900–1000 hr and 1600–1750 hr in seven healthy adults may be the low number of subjects studied.

Lower CC16 levels among subjects regularly attending chlorinated swimming pools are in accordance with the findings by our Belgian partners in the HELIOS project (Bernard et al. 2003; Carbonelle et al. 2002). These authors found that the concentrations of CC16 in trained swimmers were negatively correlated with their cumulated pool attendance. Thus, swimmers seem to have a somewhat decreased pool of CC16 in the Clara cells in the lungs. The CC16 concentration in serum reflects both the epithelial permeability and the integrity of Clara cells (Hermans and Bernard 1999). Therefore, it is conceivable that a repeated exposure to disinfecting byproducts formed by hypochlorite and organic matter (e.g., urea and sweat) in pools may decrease the CC16 secretion because of Clara cell dysfunction or damage. Thus, a possible increase in the intravascular leakage of CC16 caused by, for example, O_3_ exposure could be masked by a decrease in the production of CC16 in swimmers (Bernard et al. 2003; Carbonelle et al. 2002). That this could be the case also in our study is indicated by the tendency toward a correlation between short-term O_3_ exposure and the serum CC16 levels in non-pool visitors, but not in pool visitors, after exercise.

The levels of chlorination by-products were not measured in this study, but evidently they were high enough to affect the lung epithelium in children regularly visiting indoor pools. Because sodium hypochlorite (1% chlorine) was used as a sanitizer of the pool water, increased levels of NCl_3_ were likely to be present in the pool air. A limited number of measurements of NCl_3_ in indoor air at the swimming pool most frequently used by the swimming children in our study had been performed in 1995. Levels were similar to those reported in the same year from France by Hery et al. (1995), who identified NCl_3_ as the main component of chlorination by-products present in the air of indoor swimming pool areas. Hery et al. (1995) also reported that symptoms of irritation in the eyes and throat were correlated with the air levels of NCl_3_. Bernard et al. (2003) reported that NCl_3_ in public pools typically are in the range of 0.1–1 mg/m^3^ in air sampled 1.5 m above the water surface, that is, values similar to those reported by Hery et al. (1995) and the few Swedish measurements (Eriksson and Jacobsson, unpublished data).

## Conclusions

Our results indicate that repeated exposure to chlorination by-products in the air of indoor swimming pools has an adverse effect on the Clara cell function in children, such that the anti-inflammatory role of CC16 in the lung could be diminished. A possible role of such influence on Clara cell function in inducing pulmonary morbidity (e.g., asthma) should be further studied. The lung function parameter FEV_1_ was not adversely affected by outdoor exercise at a moderate O_3_ concentration in either pool visitors or in non-pool visitors. A possible effect of ambient O_3_ on serum CC16 levels (in nonswimming children) needs further investigation.

## Figures and Tables

**Table 1:** FEV_1_ (L/sec) and FEV_1_% predicted before (S1) and after (S2) outdoor exercise in children who do and do not regularly visit pools (mean ± SD).

Category	S1	S2	Diff S2 – S1	*p*-Value (paired *t*-test)
All (*n* = 57)				
FEV_1_	2.19 ± 0.31	2.22 ± 0.32	0.033 ± 0.061	< 0.001
FEV_1_% predicted	91.3 ± 7.2	92.7 ± 7.6	1.4 ± 2.5	< 0.001
Non-pool visitors (*n* = 34)				
FEV_1_	2.25 ± 0.32	2.29 ± 0.33	0.035 ± 0.063	0.003
FEV_1_% predicted	91.2 ± 5.6	92.6 ± 6.3	1.4 ± 2.5	0.002
Pool visitors (*n* = 23)				
FEV_1_	2.09 ± 0.27	2.13 ± 0.28	0.031 ± 0.060	0.021
FEV_1_% predicted	91.5 ± 9.1	92.9 ± 9.5	1.3 ± 2.5	0.018

Diff, difference.

**Table 2:** CC16 levels (μg/L) in plasma of children who do and do not regularly visit pools, before (S1) and after (S2) outdoor exercise (mean ± SD).

Category	S1	S2	Paired *t*-test
All (*n* = 31)	7.2 ± 2.9	7.0 ± 2.7	*p* = 0.31
Non-pool visitors (*n* = 31)	8.2 ± 2.8	8.0 ± 2.6	*p* = 0.68
Pool visitors (*n* = 20)	5.7 ± 2.4	5.3 ± 1.7	*p* = 0.14
*t*-Test pool visitors versus nonvisitors	*p* < 0.002	*p* < 0.001	

**Table 3:** Correlation between individual O_3_ exposure doses and serum CC16 concentrations in children after exercise (S2).

Category	Correlation (S2)	*p*-Value
All (*n* = 54)	0.17	< 0.21
Non-pool visitors (*n* = 33)	0.34	< 0.06
Pool visitors (*n* = 21)	−0.08	< 0.74
